# The Effect of Stress Ball on Anxiety and Pain Levels in Angiography: A Randomized Controlled Trial

**DOI:** 10.1155/2024/5049092

**Published:** 2024-08-24

**Authors:** Dilan Yüksel, Dilek Güneş

**Affiliations:** ^1^ Bingöl State Hospital, Bingöl, Turkey; ^2^ Fırat University Faculty of Health Science Department of Surgical Nursing, Elazığ, Turkey

**Keywords:** angiography, anxiety, nursing, pain, stress ball

## Abstract

**Background:** Coronary angiography, an invasive diagnostic procedure, often induces pain and anxiety in patients. Despite the potential for alleviating discomfort, the use of stress balls as a nonpharmacological intervention during angiography remains underexplored.

**Objective:** This study is aimed at investigating the impact of stress ball application on pain and anxiety levels in patients undergoing angiography.

**Methods:** This randomized controlled trial was conducted on adult patients undergoing angiography at a Cardiovascular Surgery Clinic in Eastern Turkey between January 2023 and June 2023. A total of 120 patients were randomly assigned to receive stress ball application in addition to routine care. Data collection utilized the Numerical Rating Scale (NRS), Patient Information Form, and State-Trait Anxiety Inventory (STAI).

**Results:** Analysis revealed a significantly lower increase in mean NRS posttest scores among patients in the experimental group compared to the control group (*p* < 0.05). Additionally, the mean STAI posttest score demonstrated a significant decrease (*p* < 0.05) in the experimental group. A positive and significant correlation was observed between the mean NRS and STAI posttest scores among study group patients (*p* < 0.05), indicating a reduction in anxiety levels with decreasing pain.

**Conclusion:** The application of stress balls during angiography was associated with decreased anxiety and pain levels in patients. Stress ball intervention may serve as a beneficial adjunct to pharmacological treatments. This study underscores the potential of nonpharmacological interventions in enhancing patient comfort during invasive procedures.

**Trial Registration:** ClinicalTrials.gov Identifier: NCT06131606 (http://clinicaltrials.gov).

## 1. Introduction

Coronary artery disease (CAD) ranks as one of the most significant issues affecting human health in our day and age. Despite preventive measures and therapeutic interventions, conditions such as sedentary life and increased smoking with the development of industrialization led to an increase in CADs [1, 2]. CAD develops when the coronary arteries, which ensure nourishment of the heart, become narrowed or occluded due to plaque accumulation and the heart cannot be adequately nourished and cannot perform its function. Coronary angiography (CAG) procedure provides the clearest result among CAD diagnostic methods [3, 4]. CAG is a procedure in which a radiopaque substance is administered and the vessels supplying the heart are visualized fluoroscopically with appropriate catheters in order to identify cardiovascular diseases in angiography units. After the coronary vessels of patients are monitored in terms of stenosis or occlusion with CAG, the treatment method is planned [5]. With the development of interventional cardiology, less invasive intervention, less pain, and shorter recovery time have been achieved compared to surgical cardiovascular operations [6].

There are many uncertainties related to CAG; therefore, individuals experience anxiety [7]. In addition, patients feel pain in the procedure of CAG as they feel pain in invasive procedures performed in the hospital setting [8]. For the maintenance of hemodynamic balance, there is an increase in heart rate, blood pressure, respiratory rate, and myocardial oxygen demand as a neurohormonal response to anxiety and pain [9]. It has also been reported that anxiety is a factor that exaggerates the perception of pain intensity and lowers the pain threshold [10].

Nurses play a pivotal role in managing pain and anxiety among patients, given their frequent and extended interactions with individuals undergoing medical procedures. Hence, it is imperative for nurses to possess the necessary knowledge and sensitivity and to formulate comprehensive nursing practices aimed at addressing these concerns. In addition to pharmacological interventions, nonpharmacological methods are widely employed to alleviate anxiety and pain during interventional procedures. Such methods have been reported to effectively mitigate discomfort during invasive procedures [11, 12].

For instance, Karaveli and Evirgen investigated the impact of virtual reality on anxiety and pain levels during colonoscopy and found a significant decrease in both anxiety and pain among patients exposed to virtual reality applications. These nonpharmacological interventions are favored due to their affordability, minimal risk of complications, and ease of implementation in clinical settings [13].

Among these nonpharmacological methods, the use of stress balls as a cognitive distraction technique has gained prominence in pain and anxiety management [14, 15]. Despite the documented efficacy of stress balls in reducing pain and anxiety across various contexts, their effect specifically during angiography procedures remains understudied. Therefore, this study seeks to contribute to the existing literature by examining the influence of stress ball application on pain and anxiety levels experienced by patients undergoing angiography.

The objective of this study is to investigate the impact of stress ball application on pain and anxiety levels during angiography procedures, aiming to provide insights that could inform and enhance pain and anxiety management practices in clinical settings.

### 1.1. Hypotheses of the Study

H1: Stress ball mitigates patients' pain level in angiography.

H2: Stress ball mitigates patients' anxiety levels in angiography.

## 2. Methods

### 2.1. Study Design

This controlled randomized study was carried out in the Cardiovascular Surgery Clinic (CVS) of a State Hospital in Eastern Turkey between January 2023 and June 2023.

### 2.2. Participants and Setting

**Figure 1:**
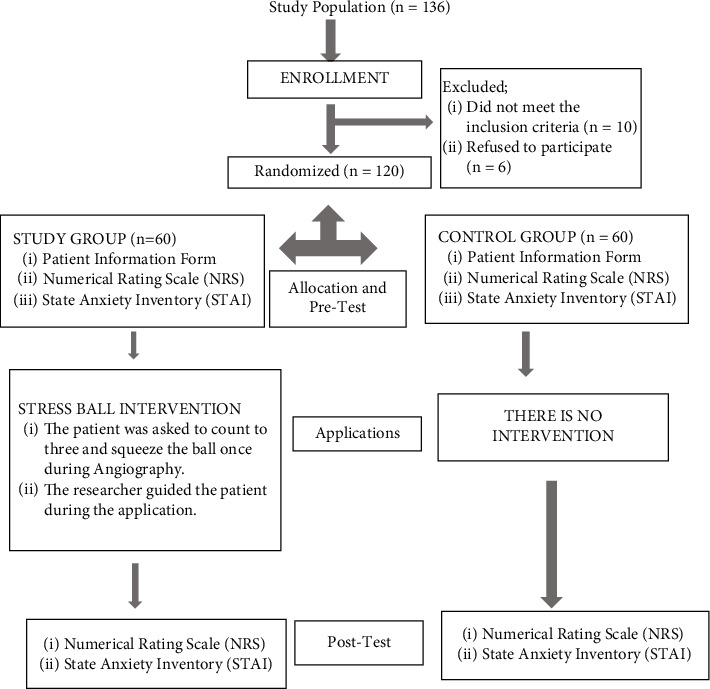
Consolidated Standards for Reporting Experiments (CONSORT) flowchart.

The research population consisted of 136 patients who underwent radial angiography in the clinic where approximately 235 angiographies are performed annually. Six patients who declined to take part in the trial and 10 patients who did not match the inclusion criteria were removed from the study. The research was finished with 120 patients (Figure 1).

The G Power 3.1.9.7 software was utilized to detect the sample size. One hundred twenty patients (60 in the control group and 60 in the study group) were determined according to the power analysis measurement with a margin of error of 0.05, an effect size of 0.7, a confidence interval of 0.95, and a power to represent the population of 95%. Guidelines from CONSORT (Consolidated Standards of Reporting Trials Statement) were used to create the research protocol [16]. Patients who met the inclusion criteria and were chosen at random from the general population made up the study sample. A computer program's algorithm was used to randomly divide the numbers from 1 to 120 into two sets [17]. The data set was blinded by the independent statistical unit. A lottery was drawn for two groups, and the first set was chosen to correspond to the study group and the second set to correspond to the control group. We followed the methods of Güneş et al. [18].

### 2.3. Inclusion Criteria

The inclusion criteria of the study are as follows: (i) no communication problems, (ii) being older than 18 years of age, (iii) having good mental health, (iv) undergoing wrist angiography (radial angiography), and (v) being willing to take part in the study.

The exclusion criterion is as follows: (i) any complication during angiography.

### 2.4. Measurements

#### 2.4.1. Patient Information Form

This form, prepared by the researcher in accordance with the relevant literature, records patient information [13–15]. In the form, characteristics such as age, marital status, gender, number of previous angiography procedures, education level, and presence of other diseases are analyzed.

#### 2.4.2. Numerical Rating Scale (NRS)

This scale, which assesses the degree of pain, is aimed at making the patient express their pain in numbers. It starts with no pain (0) and goes up to unbearable pain (10 or 100). The high level of pain reporting is known as a disadvantage. Miró, Castarlenas, and Huguet found that the validity of the NRS for pain assessment was at an acceptable level [19]. Hjermstad et al. compared the Visual Analog Scale (VAS), NRS, and Verbal Descriptor Scale (VDS) for the assessment of pain intensity in adults and found that the NRS was more understandable and easier to use than the VAS and VDS [20].

#### 2.4.3. State-Trait Anxiety Inventory (STAI)

The scale was developed by Spielberger, Gorsuch, and Lushene [21]. Its reliability and validity in our country were performed by Öner and Le Compte [22]. The scale consists of two subunits that measure anxiety separately, but in this study, the state-trait anxiety that patients feel indirectly from the stressful situation they are in will be used. A scale of 4-point Likert-type consisting of 20-item short statements measures the state-trait anxiety level of the individual. For the direct statements, the total weight score is collected throughout the evaluation and is subtracted from the total score for the reversed statements after the total weights of the direct and reversed statements have been independently determined. An unchanging and predetermined value is summed to this number, which is 50. The total score obtained from the scale ranges between 80 and 20, with a high score indicating a high anxiety level. Cronbach alpha coefficient for state-trait anxiety is 0.83 [23]. In the present research, the Cronbach alpha coefficient was ascertained to be 0.68.

### 2.5. Data Collection

The data were gathered by the first researcher between January 2023 and April 2023 by face-to-face interview method. We collected data from the patients in the study and control groups every weekday.

### 2.6. Interventions

#### 2.6.1. Study Group

A stress ball, one of the nonpharmacological methods, was used in the research. Enough stress balls were provided by the investigator before the start of the study. The stress ball is 6 cm in diameter and round and has a medium-hardness, high-quality compressible structure with sporty patterns. Angiography was performed every weekday in the morning hours. The patients were admitted to the ward, and angiography consent was obtained. We removed the patient's dressing and assisted him/her in putting on a surgical gown to protect his/her privacy. Patient Information Form, NRS, and STAI were filled out 20–25 min before the procedure. Patients in the study group were told how to use the stress ball before the procedure. The patient was then taken to the angiography unit. The stress ball was placed on the palms of the patients according to the procedure position. During the angiography, the researcher asked the patient to squeeze the stress ball once for every count of three. The angiography procedure lasted approximately 25–30 min. After the procedure, the NRS and STAI were filled out as a posttest 10 min after the patient was taken to the ward. The answers given by the patients were recorded by the researcher by marking them on the forms.

#### 2.6.2. Control Group

In the control group, the Patient Information Form, NRS, and STAI were filled out by the investigator in the clinic as a pretest. No intervention other than the clinic protocol was applied to this group. After the angiography procedure, NRS and STAI were filled out as a posttest 10 min after the patients were taken to the ward. The answers were recorded by the investigator by marking on the forms.

### 2.7. Data Assessment

The statistical significance value was accepted to be *p* < 0.05, and the data were analyzed using the SPSS (Statistical Package for Social Science) v22.0 analytical tool [24]. Data analyzed using normality test was performed using Shapiro–Wilk test, *χ*^2^tests, *t*-tests, descriptive statistics, and correlation tests.

### 2.8. Ethical Considerations

Permission was taken from the University Ethics Committee for this study (permission no. 2022/12-11). Study participants voluntarily agreed to enroll in the study. The objective of the study was clarified by the researchers, and written informed consent was sought from those who agreed to enroll in the study. Patients who participated in the study voluntarily were assured by the researchers that all their information would be kept confidential, that the obtained data would be utilized only for research purposes, and that they could opt out of the research at any time. The study was implemented in accordance with the principles of the Declaration of Helsinki.

## 3. Results

In the study group, the mean age of patients was 47.3 ± 10.4 years. Of these patients, 54.1% were female, 28.3% were high school graduates, 83.3% were married, 46.7% underwent angiography for the first time, and 60% had no comorbidities. Comparatively, the control group had an average age of 51.0 ± 13.2 years. Within the control group, 52.5% were female, 30% were high school graduates, 78.3% were married, 50% had angiography for the first time, and 50% had no comorbidities. There was no statistically significant difference between the experimental and control groups in terms of demographic characteristics (*p* > 0.05) (Table 1).

According to the posttest and pretest values of the study group, it was determined that the pain of female patients was higher than male patients, the pain of married patients was higher than single patients, and the pain of literate patients increased less than other patients. It was determined that the pain levels of patients who had not had angiography before and patients without comorbidities were higher than other patients. It was ascertained that the pain level of the study group patients increased less significantly in the posttest compared to the pain level of the control group patients (*p* < 0.05) (Table 2).

In the study group, significant decreases in state-trait anxiety were observed among male patients compared to female patients in the posttest. Additionally, single patients exhibited significantly lower state-trait anxiety levels compared to married patients, and university graduates experienced a significant reduction in state-trait anxiety compared to other educational backgrounds (*p* < 0.05). Moreover, patients in the study group who had not undergone angiography previously showed a significant decrease in state-trait anxiety compared to those who had undergone the procedure before, and patients without comorbidities experienced a significant reduction in state-trait anxiety compared to those with comorbidities (*p* < 0.05). In contrast, within the control group, a significant increase in state-trait anxiety was observed among female patients compared to male patients in the posttest. Additionally, patients who had not previously undergone angiography exhibited a significant increase in state-trait anxiety compared to others in the posttest (*p* < 0.05) (Table 3).

The study group exhibited a lesser increase in pain compared to the control group. Statistical analysis revealed a significant difference in posttest NRS values between the groups (*p* < 0.05). Furthermore, anxiety levels decreased among patients in the experimental study group, whereas they increased in the control group. Mean STAI scores showed significant differences between the groups in both pretest and posttest assessments (*p* < 0.05) (Table 4).

There was no significant relationship between NRS and STAI pretest scores (*p* > 0.05), whereas there was a significant positive relationship between posttest average scores (*p* < 0.05). It was found that the level of state-trait anxiety decreased as the pain decreased (Table 5).

## 4. Discussion

CAG is the most effective invasive diagnostic method used to examine coronary arteries, identify stenosis, and plan medical or surgical treatment. Nonpharmacologic methods are widely used as an alternative to drug treatment to reduce anxiety and pain in interventional procedures [3]. Nonpharmacologic methods include cost-effective interventions that are more widely used due to lower cost, fewer complications, and ease of clinical use [11, 25]. Stress ball squeezing, which is one of the nonpharmacologic methods, was found to be an effective way to reduce the pain and anxiety of patients [15].

In the pain assessment according to gender, it was determined that female patients experienced significantly more pain than male patients. This observation aligns with the findings of Tan and Köçkar, who evaluated pain in patients undergoing lumbar spinal surgery and similarly reported higher pain levels among female patients, suggesting a potential lower pain tolerance in women [26]. Furthermore, married patients exhibited higher pain levels compared to single patients. This disparity may be attributed to familial factors and lifestyle differences. Additionally, it was observed that literate patients experienced less pain compared to those with lower levels of education. Consistent with this, Kaya and Karagözoğlu found that illiterate patients diagnosed with lumbar disc herniation reported higher pain levels [27]. Similarly, Tan and Köçkar reported higher pain intensity among patients with lower educational levels in lumbar spinal surgery cases [26]. This underscores the significance of education in understanding and implementing pain management strategies suggested by healthcare professionals. Thus, the study findings are in line with existing literature, indicating a positive association between education level and pain reduction.

The study revealed that patients who had not undergone angiography before reported higher pain levels compared to other patients. This heightened pain experience among first-time patients is likely attributable to their lack of prior experience with the angiography procedure. Furthermore, it was observed that patients without comorbidities experienced higher pain levels compared to those with comorbidities. Interestingly, Amiri et al. conducted a meta-analysis study on pain threshold in patients with chronic pain and found that individuals with chronic pain had a lower pain threshold than healthy individuals, contrary to our study findings [28]. This disparity may be influenced by various factors such as pain beliefs, cultural teachings, and psychological and social variables impacting pain perception.

When anxiety levels of patients undergoing CAG were evaluated based on demographic characteristics before and after stress ball application, it was observed that anxiety levels decreased more among male patients in the experimental study group compared to female patients. Conversely, in the control group, anxiety levels increased more among female patients than male patients. This aligns with the findings of Türker and Bedük, who evaluated anxiety in patients undergoing CAG and found that women scored higher than men in anxiety assessments based on gender [29]. It is plausible that women's negative thoughts about the angiography procedure and their fear of the procedure contribute to their heightened anxiety levels.

When the anxiety levels of the patients were evaluated according to their marital status, the single patients' anxiety levels in the study group decreased more than married patients in the posttest. In the study by Altınbaş and Kutanis investigating the anxiety level of patients scheduled for elective surgery, the anxiety level of married patients was found to be higher [30]. Single patients' lack of responsibilities such as spouse and child care may be the reason why they experience less anxiety.

When the anxiety levels of the patients according to their educational status were analyzed, it was detected that the anxiety levels of the university graduate patients in the study group were lower than those of the patients with other educational levels in the posttest. In the study of Altınbaş and Kutanis, in parallel with our study, it was observed that the anxiety levels of the patients decreased as their education levels increased [30]. This may be related to higher university graduates being more knowledgeable or inquisitive about the procedure.

The anxiety levels of patients in the study group who had not undergone angiography before decreased more than those who had undergone angiography several times. In a study by Yıldırım and Oğuz investigating the effect of music recitals on anxiety levels in percutaneous coronary intervention, it was found that the presence of a history of CAG in the past did not affect anxiety [31]. The reason for this may be that there may be a difference in anxiety depending on whether the previous angiography experience was good or bad.

Patients in the study group who did not have any other comorbidity were found to have lower anxiety levels in the posttest in comparison to the other patients. Buldan and Kuzu examined the depression and anxiety levels of patients with chronic diseases and found that the anxiety level of patients with chronic diseases was lower [32]. Yıldırım and Oğuz also reported that the state-trait anxiety levels of patients with chronic disease were lower than those without chronic disease [31]. This difference may be due to the way the sample group perceived the disease or lack of information.

There are no studies in the literature examining the influence of stress balls on pain and anxiety levels in angiography. However, there are studies showing that stress ball decreases pain and anxiety levels in different patient groups [33–35]. It was ascertained that the pain of the study group patients increased less than the control group. Posttest pain levels revealed a significant difference between the groups. Patients' anxiety decreased in the study group and rose in the control group. The posttest and pretest mean scores of anxiety levels showed a significant difference between the groups. In a randomized controlled study conducted by Gezginci et al. on 120 patients to compare the effects of the stress ball, music, and video application on anxiety, pain, and satisfaction levels during cystoscopy, it was observed that anxiety levels and pain levels were significantly lower in the intervention groups after cystoscopy compared to the control group [33]. The study result is similar to our research findings.

Nonpharmacological methods are widely recognized for their positive effects in reducing both pain and anxiety. Indeed, our study also demonstrated a decrease in pain and anxiety levels among patients undergoing angiography, confirming our hypotheses that “Stress ball reduces the pain level of patients in angiography” and “Stress ball reduces the anxiety level of patients in angiography.”

The effectiveness of various nonpharmacological methods, including spirometer blowing, coughing technique, and stress ball squeezing, in reducing pain during intravenous catheterization procedures was evaluated by Yılmaz and Güneş. They found that patients in the spirometer blowing, coughing technique, and stress ball groups reported lower pain scores compared to the control group, indicating the effectiveness of stress ball squeezing in pain reduction [34]. Similarly, Kasar, Erzincanli, and Akbas conducted a randomized controlled study investigating the effect of stress ball use on vital signs, stress, and comfort levels in hemodialysis patients. They reported a significant decrease in stress levels in the stress ball group, while stress levels increased in the control group [35]. Additionally, Gezginci et al. demonstrated the positive effect of stress balls on pain during the extracorporeal shock wave lithotripsy (ESWL) procedure, further supporting our findings [36]. Soltani et al. in the study where they investigated the effect of using antistress balls during inferior alveolar nerve block injection in reducing the pain of patients, it was found that the pain of the patients in the experimental group decreased [37]. The methodological similarity and consistent results across these studies, characterized by pretest and posttest assessments with randomized control groups, provide further support for our study.

Furthermore, our analysis revealed a significant positive relationship between pain and anxiety posttest scores, indicating that as pain decreased, state anxiety levels also decreased. This finding is consistent with previous research by Dutucu, Özdilek, and Bektaş, who examined that the effect of virtual reality on pain and anxiety during mammography and similarly found a positive relationship between anxiety and pain scores [38]. Similarly, Ok and Kısa reported a significant positive correlation between pain and anxiety in their study. Given that high anxiety following an angiography procedure can adversely affect pain control in patients, addressing anxiety alongside pain management is crucial [10].

The results of this study align with our initial hypotheses regarding the effectiveness of stress ball intervention in reducing both pain and anxiety levels during angiography procedures. Our findings provide empirical support for the hypothesis that stress ball application decreases patients' pain levels, as evidenced by statistically significant differences in posttest pain scores between the experimental and control groups. Similarly, the observed reduction in anxiety levels among patients in the experimental group corroborates our hypothesis that stress ball intervention mitigates anxiety during angiography.

## 5. Study Limitations

The study has several limitations that warrant consideration. Firstly, the sample size was specific to the study's region and may not fully represent broader populations, limiting the generalizability of the findings. Not using patients' vital signs may be a deficiency. Additionally, while efforts were made to standardize the control group, there may have been unaccounted for variables influencing the outcomes. Moreover, the use of self-reported scales to assess pain and anxiety levels introduces subjective biases. Future research incorporating objective measures could offer a more comprehensive understanding. Furthermore, the study focused solely on stress ball intervention, omitting the exploration of potential synergistic effects with other nonpharmacological methods. Short-term follow-up assessments were conducted, lacking insights into long-term sustainability. Lastly, despite randomization efforts, inherent biases may have influenced participant selection and data analysis. Addressing these limitations could guide future research in overcoming these challenges and advancing knowledge in pain and anxiety management during angiography procedures.

## 6. Conclusion

In conclusion, this study provides valuable insights into the effectiveness of stress ball intervention as a nonpharmacological approach to pain and anxiety management in patients undergoing angiography. Our findings support the hypothesis that stress ball application reduces both pain and anxiety levels during the procedure. The positive outcomes observed align with existing literature on the beneficial effects of nonpharmacological methods in clinical settings.

## Figures and Tables

**Table 1:** Distribution of patients according to their identifying characteristics (*n* = 120).

	**Study**		**Control**		
	**n**	**%**	**n**	**%**	
Age	47.29 ± 10.4		50.96 ± 13.15		
Gender					
Female	31	54.1	33	52.5	*χ* ^2^: 0.125
Male	29	45.9	27	47.5	*p*: 0.723
Marital status					
Married	50	83.3	47	78.3	*χ* ^2^: 0.078
Single	10	16.7	13	21.7	*p*: 0.780
Education level					
Illiterate	17	28.3	13	21.7	
Literate	8	13.3	15	25.0	
Primary education	10	16.7	4	6.7	*χ* ^2^: 15.939
High school	17	28.3	18	30.0	*p*: 0.457
University	8	13.3	10	16.7	
Number of previous angiographies					
None	28	46.7	30	50.0	
1	14	23.3	9	15.0	
2	11	18.3	13	21.7	*χ* ^2^: 21.433
3	4	6.7	4	6.7	*p*: 0.372
4	2	3.3	4	6.7	
5	1	1.7	0	0	
Presence of comorbidities					
Yes	24	40.0	30	50.0	*χ* ^2^: 0.135
No	36	60.0	30	50.0	*p*: 0.457

**Table 2:** Intergroup comparison of mean NRS scores of the study and control group patients.

**NRS**	**Pretest**			**Posttest**		
	**Study (** **n** = 60**)**	**Control (** **n** = 60**)**	**Test**	**Study (** **n** = 60**)**	**Control (** **n** = 60**)**	**Test**
Gender						
Female	0.187 ± 0.535	0.125 ± 0.336	*t* : 0.528	2.250 ± 2.271	5.281 ± 1.800	*t* : −4.997
			*p* : 0.601			*p* : 0.001
Male	0.172 ± 0.539	0.069 ± 0.257	*t* : 0.902	2.000 ± 2.591	5.344 ± 2.022	*t* : −5.062
			*p* : 0.375			*p* : 0.001
Marital status						
Married	0.160 ± 0.509	0.120 ± 0.328	*t* : 0.444	2.180 ± 2.413	5.340 ± 1.791	*t* : −6.714
			*p* : 0.659			*p* : 0.001
Single	0.272 ± 0.646	0.000 ± 0.000	*t* : 1.399	1.909 ± 2.50817	5.181 ± 2.400	*t* : −2.571
			*p* : 0.192			*p* : 0.028
Education level						
Illiterate	0.055 ± 0.235	0.166 ± 0.383	*t* : −1.000	2.166 ± 2.007	5.944 ± 1.924	*t* : −4.831
			*p* : 0.331			*p* : 0.001
Literate	0.500 ± 0.925	0.000 ± 0.000	*t* : 1.528	1.375 ± 2.559	4.625 ± 1.685	*t* : −3.005
			*p* : 0.170			*p* : 0.020
Primary education	0.200 ± 0.632	0.200 ± 0.421	*t* : 0.000	1.200 ± 1.873	5.200 ± 1.932	*t* : −3.686
			*p* : 1.000			*p* : 0.005
High school	0.117 ± 0.332	0.058 ± 0.242	*t* : 0.566	2.823 ± 2.579	4.882 ± 1.763	*t* : −2.254
			*p* : 0.579			*p* : 0.039
University	0.250 ± 0.707	0.000 ± 0.000	*t* : 1.000	2.500 ± 3.251	5.625 ± 2.199	*t* : −2.328
			*p* : 0.351			*p* : 0.053
Number of previous angiographies						
None	0.178 ± 0.475	0.071 ± 0.262	*t* : 1.000	2.464 ± 2.714	5.500 ± 1.914	*t* : −4.299
			*p* : 0.326			*p* : 0.001
1	0.024 ± 0.012	0.026 ± 0.010	*t* : −0.986	1.733 ± 2.153	5.533 ± 1.767	*t* : −4.269
			*p* : 0.926			*p* : 0.001
2+	0.333 ± 0.766	0.222 ± 0.427	*t* : 0.489	1.944 ± 2.154	4.833 ± 1.977	*t* : −3.878
			*p* : 0.631			*p* : 0.001
Presence of comorbidities						
Yes	0.240 ± 0.663	0.120 ± 0.331	*t* : 0.768	2.040 ± 2.244	5.400 ± 1.848	*t* : −5.293
			*p* : 0.450			*p* : 0.001
No.	0.138 ± 0.424	0.083 ± 0.280	*t* : 0.627	2.194 ± 2.550	5.250 ± 1.947	*t* : −4.961
			*p* : 0.535			*p* : 0.001

Abbreviation: NRS, Numerical Rating Scale.

**Table 3:** Intergroup comparison of mean STAI scores of study and control group patients.

**NRS**	**Pretest**			**Posttest**		
	**Study (** **n** = 60**)**	**Control (** **n** = 60**)**	**Test**	**Study (** **n** = 60**)**	**Control (** **n** = 60**)**	**Test**
Gender						
Female	15.250 ± 4.125	14.968 ± 3.267	*t* : 1.273	10.757 + 2.326	12.060 + 3.120	*t* : −4.009
			*p* : 0.213			*p* : 0.001
Male	16.586 ± 4.526	14.862 ± 3.456	*t* : 2.317	11.928 + 2.418	13.392 + 3.480	*t* : −1.306
			*p* : 0.028			*p* : 0.202
Marital status						
Married	15.420 ± 0.412	14.960 ± 4.685	*t* : 0.640	11.100 ± 4.282	12.740 ± 6.213	*t* : −1.540
			*p* : 0.525			*p* : 0.130
Single	18.000 ± 3.820	14.727 ± 2.831	*t* : 3.655	12.181 ± 3.487	12.363 ± 3.264	*t* : −0.410
			*p* : 0.004			*p* : 0.690
Education level						
Illiterate	17.222 ± 4.505	16.777 ± 6.421	*t* : 0.258	10.333 ± 5.830	12.833 ± 5.512	*t* : −1.176
			*p* : 0.800			*p* : 0.256
Literate	15.500 ± 3.779	15.000 ± 3.295	*t* : 0.326	10.375 ± 1.846	9.750 ± 1.581	*t* : 1.488
			*p* : 0.754			*p* : 0.180
Primary education	14.700 ± 3.802	14.600 ± 2.674	*t* : 0.087	12.400 ± 4.087	16.500 ± 10.690	*t* : −1.116
			*p* : 0.933			*p* : 0.293
High school	14.647 ± 3.353	13.529 ± 2.918	*t* : 1.744	12.294 ± 3.293	12.294 ± 3.177	*t* : 0.000
			*p* : 0.100			*p* : 1.000
University	17.375 ± 2.615	14.000 ± 3.295	*t* : 2.664	10.875 ± 2.695	11.250 ± 2.434	*t* : −1.000
			*p* : 0.032			*p* : 0.351
Number of previous angiographies						
None	16.392 ± 3.774	14.107 ± 3.022	*t* : 3.680	11.035 ± 3.310	11.500 ± 3.085	*t* : −2.159
			*p* : 0.001			*p* : 0.040
1	16.058 ± 4.249	16.000 ± 2.423	*t* : 0.064	10.235 ± 5.651	14.352 ± 9.558	*t* : −1.337
			*p* : 0.950			*p* : 0.200
2+	15.166 ± 3.682	15.055 ± 6.769	*t* : 0.066	12.166 ± 3.745	12.388 ± 3.728	*t* : −0.697
			*p* : 0.948			*p* : 0.495
Presence of comorbidities						
Yes	15.541 ± 3.729	15.108 ± 5.237	*t* : 1.876	11.125 ± 3.455	13.000 ± 7.558	*t* : −1.207
			*p* : 0.317			*p* : 0.240
No.	16.108 ± 3.977	14.625 ± 2.683	*t* : 1.015	11.405 ± 4.579	12.459 ± 4.349	*t* : −1.001
			*p* : 0.033			*p* : 0.323

Abbreviation: STAI, State-Trait Anxiety Inventory.

**Table 4:** Comparison of the mean NRS and STAI scores of the control and study group patients.

**Scale**	**Study (** **n** = 60**)** **X** ± **SD**	**Control (** **n** = 60**)** **X** ± **SD**	**Test**
NRS pretest	0.180 ± 0.532	0.098 ± 0.300	*t* = 1.000 *p* = 0.321
NRS posttest	2.131 ± 2.411	5.311 ± 1.893	*t* = −7.165 *p* = 0.001
STAI pretest	15.885 ± 3.860	11.295 ± 4.144	*t* = 5.735 *p* = 0.001
STAI posttest	14.918 ± 4.390	12.672 ± 5.772	*t* = 2.415 *p* = 0.019

Abbreviations: NRS, Numerical Rating Scale; STAI, State-Trait Anxiety Inventory.

**Table 5:** Examination of the relationship between NRS and STAI posttest and pretest scores of the study group patients.

**Scale**	**n**	**Test**
Pretest NRS-STAI	60	*r* = 0.083; *p* = 0.524
Posttest NRS-STAI	60	*r* = 0.257; *p* = 0.046

Abbreviations: NRS, Numerical Rating Scale; STAI, State-Trait Anxiety Inventory.

## Data Availability

The data used to support the findings of this study are included in the article. Further requests for data, after publication of this article, can be directed to the corresponding author.
